# CDK-inhibitor independent cell cycle progression in an experimental haematopoietic stem cell leukaemia despite unaltered Rb-phosphorylation

**DOI:** 10.1038/sj.bjc.6690768

**Published:** 1999-11

**Authors:** R Huss, S Theis, H J Deeg

**Affiliations:** Institute of Pathology, University of Munich, Thalkirchner Str. 36, Munich D-80337, Germany; Division of Clinical Research, Fred Hutchinson Cancer Research Center and the Department of Medicine, University of Washington, 1100 Fairview Ave N, PO Box 19024, Seattle, WA 98109-1024, USA

**Keywords:** haematopoiesis, CD34, MHC class II, cell cycle, Rb-phosphorylation

## Abstract

A CD34-negative haematopoietic progenitor cell line, D064, derived from canine bone marrow stromal cells is able to differentiate into haematopoietic progenitors under the influence of growth factor-mediated signalling. While differentiating, these cells eventually start to express MHC class II molecules (DR homologues) on their surface. The stable transfection of the fibroblast-like wild-type cells with retroviral constructs containing the cDNA for the canine MHC class II DR-genes (DRA and DRB) induces a change in morphology, accelerates cell cycle progression and leads to a loss of anchorage-dependent growth. Transfected cells show features of an immature stem cell leukaemia, such as giant cell formation. In wild-type D064 cells the accumulation of the cyclin-dependent kinase inhibitor (cdki) p27^kip-1^ induces differentiation, which is dependent upon signalling via the ligand for the tyrosine kinase receptor c-kit (stem cell factor). DR-transfected cells instead apparently grow independently of any growth factor-mediated signals and express high levels of the cdkis p27^kip-1^ and especially p21^waf-1/cip-1^, concurrently with accelated cell cycle progression. In contrast to the overexpression of cdkis and despite accelerated cell cycle progression, the expression of the G2/M phase transition kinase p34^cdc2^ is significantly reduced in DR-transfected and transformed cells as compared to the haematopoietic wild-type cell line D064. This might suggest a possible alternative cell cycle progression pathway in this experimental stem cell leukaemia by by-passing the G0/G1 phase arrest, although retinoblastoma (Rb)-phosphorylation remains unaltered. These results provide evidence that mechanisms normally controlling the cell cycle and early haematopoietic differentiation are disrupted by the constitutive transcription and expression of MHC class II genes (DR) leading to a progression and growth of this experimental stem cell leukaemia independent from cell cycle controlling regulators such as p27 and p21. © 1999 Cancer Research Campaign


*British Journal of Cancer* (1999) **81**(5), 808–813
© 1999 Cancer Research Campaign
Article no. bjoc.1999.0768

### CDK-inhibitor independent cell cycle progression in an experimental haematopoietic stem cell leukaemia despite unaltered Rb-phosphorylation

**R Huss[1], S Theis[1] and HJ Deeg[2]**

[1]Institute of Pathology, University of Munich, Thalkirchner Str. 36, D-80337 Munich, Germany; [2]Division of Clinical Research, Fred Hutchinson Cancer Research Center and the Department of Medicine, University of Washington, 1100 Fairview Ave N, PO Box 19024, Seattle, WA 98109-1024, USA

**Summary** A CD34-negative haematopoietic progenitor cell line, D064, derived from canine bone marrow stromal cells is able to differentiate into haematopoietic progenitors under the influence of growth factor-mediated signalling. While differentiating, these cells eventually start to express MHC class II molecules (DR homologues) on their surface. The stable transfection of the fibroblast-like wild-type cells with retroviral constructs containing the cDNA for the canine MHC class II DR-genes (DRA and DRB) induces a change in morphology, accelerates cell cycle progression and leads to a loss of anchorage-dependent growth. Transfected cells show features of an immature stem cell leukaemia, such as giant cell formation. In wild-type D064 cells the accumulation of the cyclin-dependent kinase inhibitor (cdki) $p27^{kip\text{-}1}$ induces differentiation, which is dependent upon signalling via the ligand for the tyrosine kinase receptor c-kit (stem cell factor). DR-transfected cells instead apparently grow independently of any growth factor-mediated signals and express high levels of the cdkis $p27^{kip\text{-}1}$ and especially $p21^{waf\text{-}1/cip\text{-}1}$, concurrently with accelated cell cycle progression. In contrast to the overexpression of cdkis and despite accelerated cell cycle progression, the expression of the G2/M phase transition kinase $p34^{cdc2}$ is significantly reduced in DR-transfected and transformed cells as compared to the haematopoietic wild-type cell line D064. This might suggest a possible alternative cell cycle progression pathway in this experimental stem cell leukaemia by by-passing the G0/G1 phase arrest, although retinoblastoma (Rb)-phosphorylation remains unaltered. These results provide evidence that mechanisms normally controlling the cell cycle and early haematopoietic differentiation are disrupted by the constitutive transcription and expression of MHC class II genes (DR) leading to a progression and growth of this experimental stem cell leukaemia independent from cell cycle controlling regulators such as p27 and p21. © 1999 Cancer Research Campaign

**Keywords**: haematopoiesis; CD34; MHC class II; cell cycle; Rb-phosphorylation

Haematopoiesis is a process that requires haematopoietic progenitor cells to differentiate into mature cells of multiple lineages. The starting point of this maturation is presumably a multipotential stem cell, which has the ability of self-renewal and differentiation into all lineages. The regulation of differentiation depends on a variety of cellular and soluble factors provided in part by the so-called marrow microenvironment consisting of stromal elements (Tavassoli and Friedenstein, 1983; Huss et al, 1995*a*). Other factors may function in an autocrine fashion, and cell–cell interactions are also of pivotal importance. During differentiation, many transcriptional events occur in a way so that they provide the essential gene products for a certain stage of differentiation (Dorshkind, 1990; Eaves et al, 1991).

Cell division in eukaryotic cells is further controlled by sequencial activation of a family of serine-threonine protein kinases, known as cyclin-dependent kinases (cdks). cdks regulate progression through various key transitions in the cell cycle of all eukaryotes studied so far. Two distinct cdks, cdk4 or cdk6 and cdk2, control G1 progression and entry into S phase. Inactivation of either kinase leads to cell cycle arrest and withdrawal from the mitotic cycle (Roberts et al, 1994; Sher and Roberts, 1995).

*Received 10 March 1999*
*Revised 6 May 1999*
*Accepted 7 May 1999*

*Correspondence to:* R Huss

In recent years, a growing number of proteins, named collectively as cyclin-dependent kinase inhibitors (cdkis) were characterized as negative regulators of G1 cdks. Three members of the cdki family, $p21^{waf\text{-}1/cip\text{-}1}$, $p27^{kip\text{-}1}$ and $p57^{kip\text{-}2}$ contain a conserved domain at its N-terminal end that is both necessary and sufficient for inhibition of G1 cyclin–cdk complexes. In addition, p21 also binds to the DNA polymerase δ progression factor, proliferation cell nuclear antigen (PCNA), forming quaternary complexes containing a cyclin, a cdk and PCNA (Li et al, 1994; Waga et al, 1994). The PCNA binding activity is not observed with p27 or p57 (Luo et al, 1995). cdkis are considered versatile negative regulators of cell proliferation, likely in a cell-type specific manner (Elledge et al, 1994). They also have been implicated as targets of differentiation-inducing agents, for example, induction of p21 by the vitamin D receptor in myelomonocytic cells (Liu et al, 1996). There is also evidence supporting a role of cdkis in promoting differentiation since ectopic expression of either p21 or p27 in myelomonocytic cells induce both cell cycle arrest and induction of specific markers indicative of differentiation (Elledge and Harper, 1994; Huss et al, 1995*b*).

We established a CD34-negative, canine progenitor cell line, D064, derived from the canine marrow microenvironment (Huss et al., 1995c). These D064 cells usually grow adherent and show the typical fibroblast-like morphology of stromal cells. They support haematopoiesis in long-term marrow cultures, but are also able to differentiate into cells with characteristics of haematopoietic progenitors (Huss et al, 1995*d*). This differentiation towards lineage commitment can be induced by the addition of stem cell factor (SCF). On the contrary, interleukin-6 (IL-6) rather promotes proliferation and is counteracting differentiation (Huss et al, 1995*a*). Both factors, SCF as well as IL-6, are produced by D064 cells themselves (Huss et al, 1994). During differentiation, D064 cells partly lose their ability to grow adherent and morphologically resemble early haematopoietic progenitors. At this stage, D064 wild-type (wt) cells express $p27^{kip-1}$. p27 values decline during IL-6-mediated proliferation or treatment with an anti-SCF antibody (Huss et al, 1995*b*). Furthermore, D064 cells start to transcribe and express CD34 and eventually express MHC class II (DR) antigens as differentiation markers during SCF-mediated differentiation. The expression of MHC class II antigens is apparently of pivotal importance for a functional haematopoiesis in the dog in vitro and in vivo (Deeg et al, 1994). If anti-MHC class II monoclonal antibodies, directed at non-polymorphic determinants, are administered in vitro or in vivo, haematopoietic reconstitution is inhibited (Greinix et al, 1991). Similarly the presence of anti-class II monoclonal antibodies in long-term cultures and colony formation assays inhibit haematopoiesis in vitro (Hong et al, 1995). But the addition of exogenous SCF prevents or overcomes this inhibition both in vivo and in vitro at least in part by preventing apoptosis in a subpopulation of adherent and non-adherent cells (Hong et al, 1995).

Here we examined the effect of MHC class II molecules (DR) when constitutively expressed in a CD34-negative haematopoietic progenitor cell line. Those D064 cells were transfected with canine MHC class II (DRA and DRB) genes to investigate the effect on cell biology. As shown previously, DR-expressing cell clones show cellular transformation and tumour growth in mice, similar to an immature stem cell leukaemia (Huss et al, 1997), suggesting a disruption of mechanisms normally controlling the cell cycle. In this study we wanted to further dissect the molecular events involved in such an alternative cell cycle progression pathway, which might possibly play a role in leukaemic transformation.

### MATERIAL AND METHODS

### Cell lines

The stroma-derived cell line D064 was established from canine marrow propagated in long-term cultures and by selecting colony-forming-unit (CFU)-fibroblast as described elsewhere (Huss et al, 1995*c*). These fibroblast-like cells function as stromal cells by supporting haematopoiesis in long-term culture-initiating assays (LTC-IC), but are also able to differentiate into cells with haematopoietic characteristics. The adherent growing fibroblast-like D064 cells generally do not express detectable levels of differentiation markers, such as CD34 or MHC class II antigens (DR), but transcribe the mRNA for various growth factors (Huss et al, 1994). However, differentiation can be induced by SCF and D064 cells start to express CD34 and MHC class II-molecules (DR) on their surface. The cells then acquire a tendency to grow in a non-adherent fashion and resemble morphologically and by immunohistocytochemistry resemble haematopoietic progenitor cells.

### Experimental stem cell leukaemia

As described already elsewhere, the CD34-negative haematopoietic progenitor cell line D064 was stably transfected with retroviral constructs containing the open reading frame of DRA and DRB (Huss et al, 1997). The selection marker resistent cell clones were further selected for high levels of DR-expression.

### Immunohistochemistry

The expression of p27 in transfected and non-transfected cells was evaluated by immunophenotyping using a monoclonal antibody directed against $p27^{kip-1}$. D064 cells and the $DR^+$ clone no. 14 were grown in chamber slides (NUNC) and fixed with ethanol. Cells were then permeabilized using Triton X-100 and stained with the appropriate primary antibody (Santa Cruz). Unspecific staining was blocked using the Universal powerblock (Biogenex) in a one-tenth dilution for 15 min prior to using the primary antibody. Immunodetection staining was carried out with the Multilink detection kit (Biogenex) using alkaline-phosphatase conjugated streptavidin and Fast Red.

### Western blot analysis

A total of $5 \times 10^6$ transfected (clone no. 14) or non-transfected D064 cells were grown with or without SCF (100 ng $ml^{-1}$) for 2, 24, 48 and 96 h and solubilized with lysis buffer. Equal amounts of protein were loaded in each lane of the gel as determined by quantitative spectrophotometry. The gels were blotted overnight on a nitrocellulose membrane (Amersham) by electrophoretic transfer at 4°C. Non-specific antibody binding sites were blocked with 1 mg $ml^{-1}$ instant, non-fat dry milk in phosphate-buffered saline (PBS). The filter was incubated with anti-p27, anti-p21, anti-p34 (Santa Cruz) and anti-phosphotyrosine antibody (PY-20, Pharmingen) as the primary antibody. After washing, the secondary alkaline-phosphatase detection antibody (Tago, Burlingame, CA, USA) was added. After 1-h incubation, the membrane was again thoroughly washed and incubated in chemiluminescence substrate (CSPD) solution (Clontech Laboratories) for 5 min. The membrane was then placed on an X-ray film for 3 s (Firpo et al, 1994; Nourse et al, 1994). In one set of the experiments the lysed cell preparation was immunoprecipitated with an anti-Rb monoclonal antibody (Oncogen) as described elsewhere (Haefner et al, 1994; Weissinger et al, 1997).

### Cell cycle analysis

At various time points, monolayers of transfected and non-transfected D064 cells were treated with trypsin (0.05%) for 15 min at 37°C. Cells were washed with 1 × Hanks balanced salt solution (HBSS) and processed for DNA content. Briefly, $1 \times 10^6$ cells were resuspended in 250 µl of buffer A (10 mmol $l^{-1}$ Tris–HCl, pH 7.5; 20 mmol $l^{-1}$ sodium chloride, 20 mmol $l^{-1}$ magnesium chloride). After 5 min on ice, 250 µl of buffer B was added (buffer A containing 1% NP-40) and the mixture was incubated for another 5 min on ice. Then DNAase-free RNAase at a final concentration of 2.5 µg was added and nuclei were incubated at 37°C for 30 min. Finally, 500 µl of propidium iodide (PI) solution (1 × HBSS containing 100 µg $ml^{-1}$ PI (Sigma), 0.1% Triton X-100 (USB), fetal bovine serum (HyClone)) was added to each tube and were left in the dark at 4°C for 1 h before reading. The DNA content was determined by FACScan analysis using LYSIS II software (Becton Dickinson). Cell-cycle analysis was performed with the Multicycle computer program after analysing the data by Reproman software (Fine Facts Software, Seattle, WA, USA).

© 1999 Cancer Research Campaign 

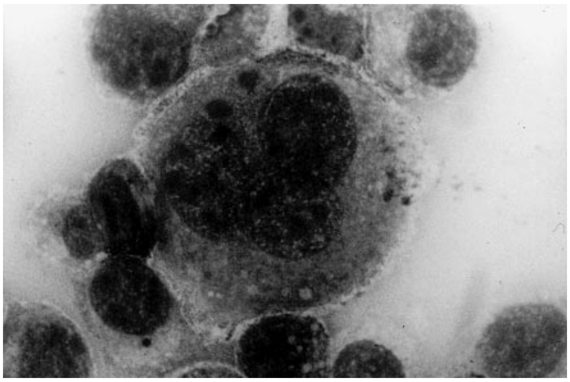

**Figure 1** Cytospin preparation of a giant cell of a DR-transfected and DR-expressing cell clone, originally derived from the parental D064 cell line

**Table 1** Number of cells (in %) in different phases of the cell cycle comparing non-transfected wt D064 cells and the DR-transfected clone no. 14

| | G0/G1 | S phase | G2 |
|---|---|---|---|
| wt D064 | 78 ± 7 | 10 ± 4 | 12 ± 4 |
| DR+ clone no. 14 | 42 ± 8 | 19 ± 5 | 39 ± 6 |

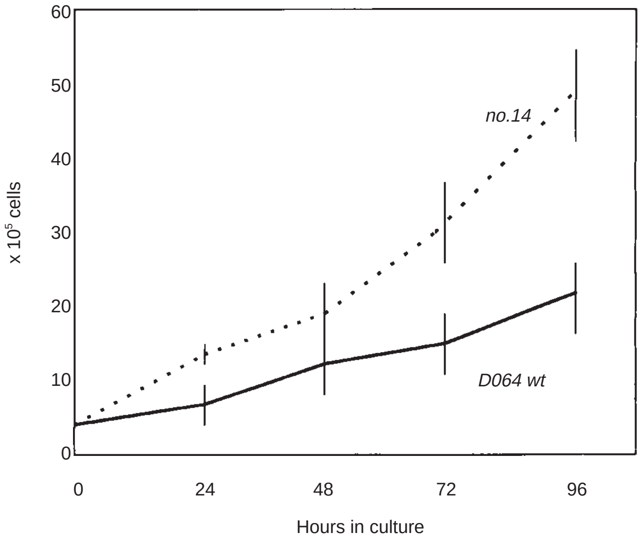

**Figure 2** Growth curve of D064 wildtype (wt) cells and DR-transfected cells (no. 14). D064 wt cells show a log phase between 0 and 48 h in cultures with a consecutive lag phase (contact inhibition) between 72 and 96 h. Number 14 cells present an accelerated cell growth with no apparent contact inhibition

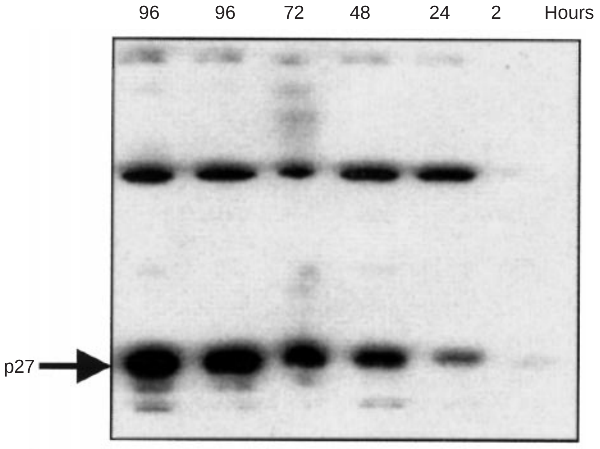

**Figure 3** Western blot analysis of $p27^{kip-1}$ expression during haematopoietic differentiation showing accumulation of $p27^{kip-1}$. After 24 h of serum starvation, SCF (100 ng $ml^{-1}$) was added to D064 cells in vitro for 96 hours. The left lane (96 h) shows the $p27^{kip-1}$ expression without any exogenous SCF, suggesting the production of SCF in an autocrine or paracrine manner, which also leads to a spontaneous accumulation of p27 and increased number of D064 cells arrested in G0/G1 phase of the cell cycle

### RESULTS

### Transfection of D064 cells

Parental CD34-negative D064 cells were transfected sequentially with the genes for canine DRA and DRB. The cDNA was contained in retroviral vectors using neomycin-phosphotransferase and L-histidin-dehydrogenase respectively as selection markers (Miller and Rosman, 1989). Transfectants were selected in the appropriate selection media. D064 cells, which were transfected only with the plasmid without any insert or with the inserts in the reverse orientation were used as controls. After selecting for antibiotic-resistant cell populations, transfected cells were sorted for high DR expression. Cell clones were established, which either contained both DRA and DRB genes or expressed high levels of DR on the surface. Control clones and non-transfected D064 clones did not transcribe any message for MHC class II (Huss et al, 1997). $DR^+$ clones were further propagated and used for the experiments (Huss et al, 1997). The transfected $DR^+$ cells lost their ability for contact inhibition when reaching confluency in culture. They also lost in part their ability to grow adherent and are predominately smaller than the non-transfected D064 cells. But as shown in Figure 1, MHC class II transfected cells though even sometimes show giant cell formation, suggesting a disruption of events controlling the cell cycle.

### Cell cycle progression of transfected D064 cells

Transfected cells are smaller than untransfected parental cells and do not require anchorage-dependent growth. $DR^+$ transfectants not only showed changes in their morphology as compared to the D064 wildtype, but also showed alterations of cell cycle progression. $DR^+$-transfected cell clones displayed a doubling time of 14.5 h as compared to approximately 48 h in parental DR–D064 cells. Accordingly, the analysis of the cell cycle showed that the number of $DR^+$-transfected cells in G2/M phase was almost twice as high as compared to the non-transfected controls over time (Table 1). This change was also reflected in the cumulative number of cells in culture with increasing time. While the wt D064 cells showed the typical pattern of lag and log-phase (0–48 h) with a growth inhibition at the confluent phase (72–96 h) (Figure 2), the DR-transfected cells showed a linear growth pattern with no significant stationary phase (not shown). The transfection-induced changes were seen only with constructs carrying the open reading frames of DRA and DRB in the correct orientation. Constructs with DRA and DRB in reverse orientation or vectors without insert did not differ from the parental cells (Huss et al, 1997).

*© 1999 Cancer Research Campaign*

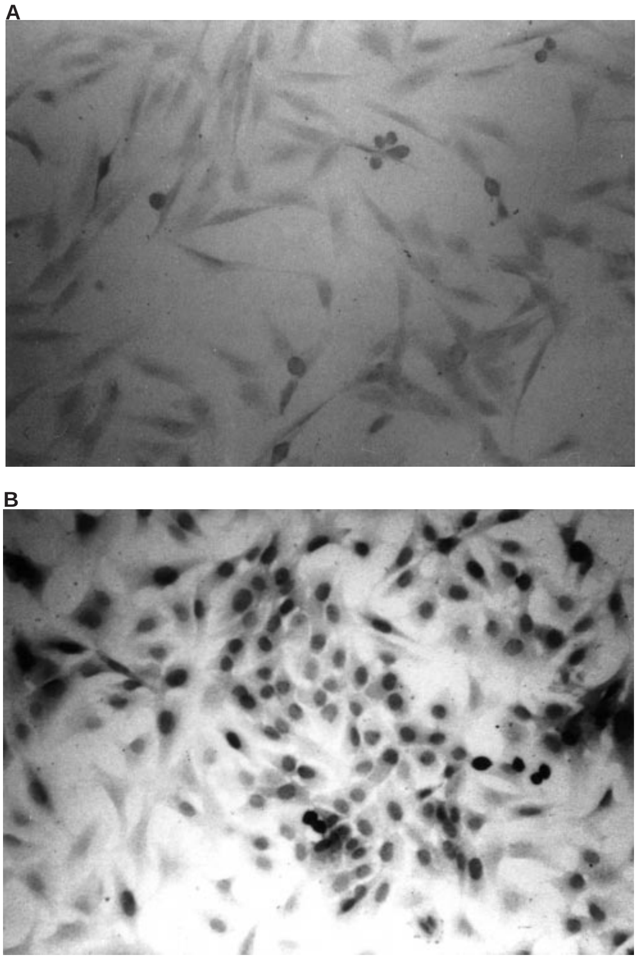

**Figure 4** **(A)** Immunophenotyping for the expression of p27$^{kip-1}$ in the parental D064 cell line. The wt cells show the typical feature of adherent-growing, fibroblast-like cells. Only few, non-adherent, differentiating cells show p27$^{kip-1}$ expression (Huss et al, 1995*b*). (**B**) Immunophenotyping for the expression of p27$^{kip-1}$ in a DR-transfected, DR-expressing cell clone. While the transfected cell clone is smaller than the parental wt cell line D064, almost all of these transformed cells show a nuclear p27$^{kip-1}$ expression

### Growth factor dependent p27$^{kip-1}$ expression

After 24 h of serum starvation D064 cells were treated with SCF for 2, 24, 48 and 96 h. p27$^{kip-1}$ accumulation was seen with increasing time (Figure 3). Also D064 cells, which were not treated with SCF at any time, show an equally high expression level of p27 after 96 h, suggesting a significant production of endogenous SCF in an autocrine or paracrine manner (Huss et al, 1994). The accumulation of p27 in D064 cells correlated with the increasing number of cells, arrested in G0/G1 phase of the cell cycle (Table 1).

### Immunophenotyping for p27$^{kip-1}$

Wt D064 cells and DR-transfected cell clones were immunostained for p27 expression after 24 h of SCF treatment. The untransfected wt D064 cells hardly showed any p27 expression and possibly only those which underwent differentiation (Figure 4A). In contrast, almost all DR$^+$ cells (clone no. 14) showed a more or less strong p27 expression, especially in the smaller cells (Figure 4B).

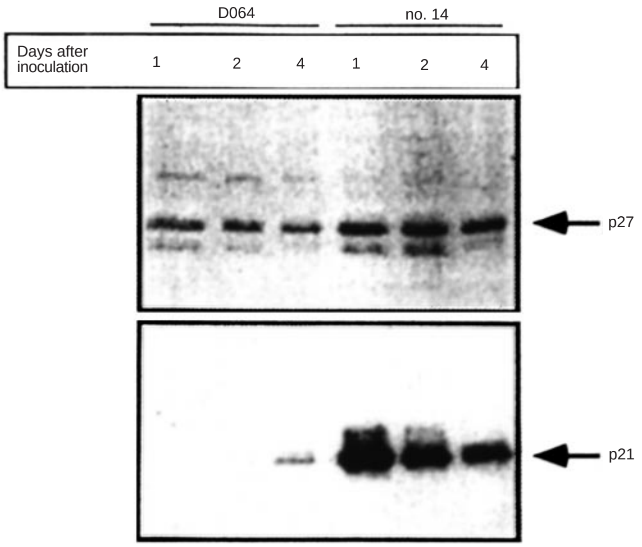

**Figure 5** Western blot analysis of p27$^{kip-1}$ and p21$^{cip-1/waf-1}$ cdk expression in growing wt D064 cells and the DR-transfected clone no. 14 at various time points after inoculation into tissue culture flasks. While p27 expression accumulates over time in the parental cell line (D064) (also see Figure 3) and p21 expression can only be observed after 4 days in culture, both cdks are overexpressed in the transformed cell clone (no. 14)

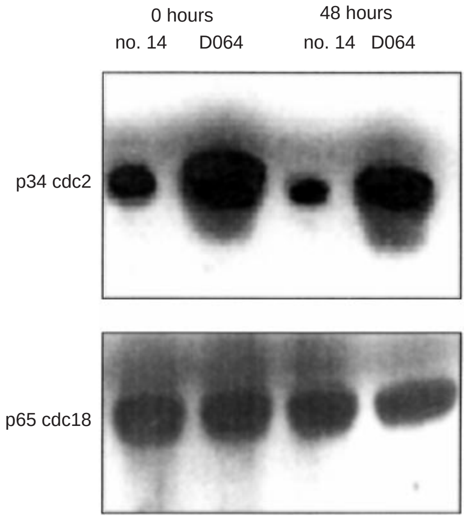

**Figure 6** p34$^{cdc2}$ expression in wt D064 cells and the DR-transfected cell clone no. 14 was performed after 48 h in culture. DR-transfected cells expressed low levels of p34 at 0 h as well as 48 h as compared to the wt cell line D064, although DR-transfected cells show a faster cell cycle progression time. P65 cdc18 remains unaltered during haematopoietic differentiation and serves as a loading control (equal amounts of proteins was loaded)

### Expression of p27 and p21

Both wt D064 and DR-transduced D064 cells were placed in tissue culture flasks on day 0 at a concentration of $5 \times 10^6$ cells ml$^{-1}$. They were grown until they reached confluency on day 4. Using equal amounts of protein for Western blot analysis, p27 was expressed over time in the spontaneously differentiating D064 cells. However, the DR$^+$ cell clones (e.g. clone no. 14) expressed elevated levels of p27 despite a higher proliferation rate. D064 cells also showed only detectable levels of p21 on day 4 during the

*© 1999 Cancer Research Campaign*

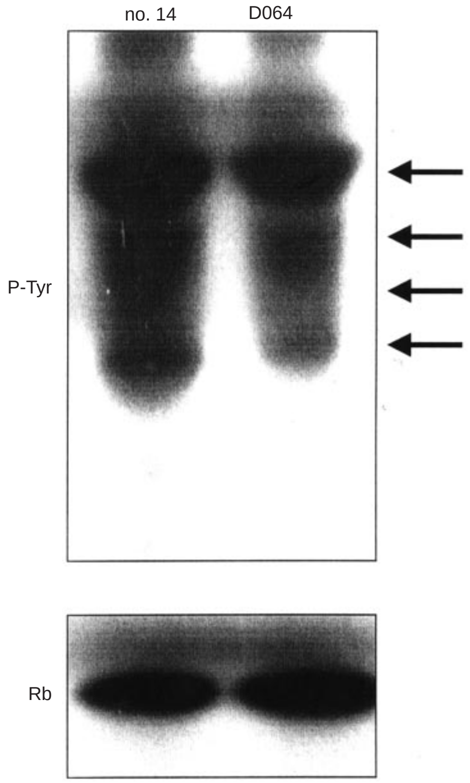

**Figure 7** Unaltered phosphorylation of the Rb protein in wt D064 cells and DR-transfected clone no. 14, showing a molecular weight of the phosphorylated isoforms from 105 to 113 kDa. At the bottom a loading control of a silver-stained sodium dodecyl sulphate gel electrophoresis after immunoprecipitation for Rb confluent phase in culture. But the DR-transfected cell clone (no. 14) overexpressed p21 from the beginning (day 1), and p21 values declined slightly over time (Figure 5).

### Expression of $p34^{cdc2}$

A comparative evaluation of $p34^{cdc2}$ expression in wt D064 cells and the DR-transfected cell clone no. 14 was performed after 48 h in culture. Figure 6 reveals that $p34^{cdc2}$ expression was reduced at 0 and 48 h as compared to the wt cell line D064, although DR-transfected cells showed increased expression of cdkis. $P65^{cdc18}$ remains unaltered during haematopoietic differentiation.

### Phosphorylation of Rb

The key element of cell cycle transition is the phosphorylation of the Rb protein, finally showing a molecular weight of the phosphorylated isoform from 105 to 113 kDa. Despite an acceleration of the cell cycle and overexpression of cdkis in DR-transfected cells, the phosphorylation of the Rb-protein remained unaltered, as compared to the untransfected wt cells (Figure 7).

### DISCUSSION

The marrow stroma-derived, CD34-negative cell line D064 usually grows adherent as fibroblast-like cells with a constant pattern of growth. When cells reach confluency in culture, growth is inhibited by a mechanism yet to be defined (Rubin et al, 1990). D064 cells can be induced to differentiate by SCF. If SCF is added to D064 cell cultures, D064 cells start to round up, lose their ability to adhere and most notably start to express differentiation antigens such as CD34 and eventually MHC class II (DR) (Huss et al, 1995*c*). Here we transfected D064 cells with retroviral constructs containing the MHC class II genes (DR). The majority of $DR^+$-transfected cells appear to be smaller than the adherent growing fibroblast-like parental cell line D064, although giant cell formation can occur. They also rounded up and lost in part their ability to grow adherent. In addition, the transfected $DR^+$ cells displayed significant changes in their growth pattern. The $DR^+$ transfectants had a significantly shortened division time as compared to parental D064 cells (14.5 vs 48 hours) and no longer showed contact inhibition in culture.

Transfection induced expression of MHC class II (DR) in the D064 cell was associated with cellular transformation in vitro and tumour forming ability in vivo (Huss et al, 1997). It appeared therefore, that the transformation ablated the growth controlling mechanisms as required for controlled growth in vitro and in vivo (Shin et al, 1975). Since the observed transformational events are not associated with any kind of retroviral signalling (mock transfection does not yield transformation), but rather relates to an induction of MHC class II (DR) expression, although initiated by the retroviral vector driven MHC class II gene (DR), expression (Glimcher and Kara, 1992). The fact that MHC class II expression continued to be present only in cell clones that had been specifically transfected with MHC class II genes, further suggests that the observed changes were related to specific expressed genes rather than due to the vector or insertional mutagenesis, since all other control transfectants remained unaltered. The expression of DR enables the cells to receive and transmit signals via MHC class II antigens (Lane et al, 1990), a signal transduction pathway yet unknown. Nevertheless, it is possible that MHC class II expression is just one parameter correlated with the transformation process. The overexpression of MHC class II in early haematopoietic stem cells parallels a specific signal transduction pathway in the transduced cells which actually leads to a cellular transformation. We have shown that SCF induces CD34, transforming growth factor β and $p27^{kip-1}$ transcription in the parental D064 cell line (Huss et al, 1995*e*). While the expression of certain factors during differentiation and proliferation is strictly regulated in the parental cell line, the $DR^+$-transfected cell clones have lost the ability to regulate those functions due to the transformational event. As demonstrated in other tumours (Elledge et al, 1996), the overexpression of the cdkis p27 and p21 was also seen in DR-transfected cells. cdkis apparently fail to control the cell cycle, suggesting the possibility that the overexpression of p27 and p21 was due to the accumulation of a dysfunctional mutant, as described for p53 (Kamp, 1995). This still has to be analysed in the DR-transfected cells, although p21 or p27 mutants have not been described so far. Nevertheless, there might be a disruption of forming the complex between cdks, cyclins, PCNA and their inhibitors or it exists as an alternative pathway bypassing the known cell cycle regulating mechanisms. The low expression or even down-regulation of $p34^{cdc2}$ as a downstream regulator of the cell cycle progression provides even more evidence for such a ‘bypass hypothesis’, especially in view of the unaltered phosphorylation pattern of the Rb protein.

The exact mechanisms of transformation of the DR-transfected, CD34-negative D064 cells are yet not known. Although there is a correlation between MHC class II expression and transformation,

 © 1999 Cancer Research Campaign we can not exclude the possibility that other molecular events involved in signal transduction pathways between any transmembrane signalling and nuclear activation might contribute to the transformation process, suggesting an alternative cell cycle progression pathway as known for most 'normal' prokaryotic and eukaryotic cells.

In summary, we have shown that the transfection and subsequent expression of differentiation antigen, such as MHC class II genes in haematopoietic progenitor cells, can induce cellular transformation and leads to a dysregulation of cell cycle control.

### ACKNOWLEDGEMENTS

We would like to thank Jose S de Oliveira, Cyntia A Hoy, Cassandra Beckham and Juliet Kniley for their excellent technical assistance and helpful discussions. We are also grateful to Eduardo S Firpo and James M Roberts for their advice and stimulating discussions regarding the cell cycle and cdk inhibitor analysis. This work was supported in parts by grants CA 31787, CA 18221 and CA 18029. R Huss was supported by a grant from the Deutsche Forschungsgemeinschaft, Bad Godesberg, Germany, and the International José Carreras Leukemia Foundation, Barcelona, Spain.

### REFERENCES

Deeg HJ, Beckham C, Huss R, Myerson D, Greinix H, Appelbaum FR, Graham T, Schuening F and Storb R (1994) Rescue from anti-MHC class II antibody-mediated marrow graft failure by c-Kit ligand. *Blood* **83**: 2352–2357

Dorshkind K (1990) Regulation of hematopoiesis by bone marrow stromal cells and their products. *Ann Rev Immunol* **8**: 111–121

Eaves CJ, Cashman JD, Kay RJ, Dougherty GJ, Otsuka T, Gaboury LA, Hogge DE, Landsdorp PM, Eaves AC and Humphries RK (1991) Mechanisms that regulate the cell cycle of very primitive hematopoietic cells in long-term human marrow cultures. II. Analysis of positive and negative regulators produced by stromal cells within adherent layer. *Blood* **78**: 110–116

Elledge SJ and Harper JW (1994) Cdk inhibitors: on the threshold of checkpoints and development. *Curr Opin Biol* **6**: 847–849

Elledge SJ, Winston J and Harper JW (1996) A question of balance: the role of cyclin-kinase inhibitors in development and tumorigenesis. *Trends Cell Biol* **6**: 388–394

Firpo ES, Koff A, Solomon MJ and Roberts JM (1994) Inactivation of a Cdk2 inhibitor during Interleukin 2-induced proliferation of human T-lymphocytes. *Mol Cell Biol* **14**: 4899–4906

Glimcher LH and Kara CJ (1992) Sequences and factors: a guide to MHC class II transcription. *Ann Rev Immunol* **10**: 13–21

Greinix HT, Ladiges WC, Graham TC, Maslan S, Raff RF, Sandmaier BM, Appelbaum FR, Schuening FG, Deeg HJ and Storb R (1991) Late failure of autologous marrow grafts in lethally irradiated dogs given anti-class-II monoclonal antibody. *Blood* **78**: 2131–2137

Haefner S, Adler HS, Mischak H, Janosch P, Heidecker G, Wolfman A, Pippig S, Lohse M, Ueffing M and Kolch W (1994) Mechanisms of inhibition of Raf-1 by protein kinase A *Mol Cell Biol* **14**: 6696–6703

Hong DS, Beckham C, Huss R, Lee JW, Hockenbery D, Ledbetter JA and Deeg HJ (1995) MHC class II mediated inhibition of hemopoiesis in long-term marrow cultures involves apoptosis and is prevented by c-kit ligand. *Blood* **86**: 3341–3346

Huss R, Hong DS, Storb R and Deeg HJ (1994) Alteration of growth factor expression in marrow stromal cells by MHC class II crosslinking. In: *Molecular Biology of Hematopoiesis, vol. 3* Abraham N, Shadduck RK, Levine AS and Takaku T (eds), pp. 719–725. Intercept Andover, UK

Huss R, Hoy CA and Deeg JH (1995*a*) Contact- and growth factor-dependent survival in a canine marrow-derived stromal cell line. *Blood* **85**: 2414–2421

Huss R, Firpo ES and Deeg HJ (1995*b*) Stem cell factor induced expression of p27 kip-1 during hematopoietic differentiation. *Exp Mol Pathol* **63**: 123–130

Huss R, Hong DS, Beckham C, Kimball L, Myerson DH, Storb R and Deeg HJ (1995*c*) Ultrastructural localization of stem cell factor in canine marrow-derived stromal cells *Exp Hematol* 195; **23**: 33–40

Huss R, Hong DS, McSweeney PA, Hoy CA and Deeg HJ (1995*d*) Differentiation of canine bone marrow cells with characteristics from an adherent stromal cell precursor. *Proc Natl Acad Sci USA* **92**: 748–752

Huss R, Hoy CA and Deeg HJ (1995*e*) Stroma derived hemopoietic progenitors: cell cycle dependent proliferation and differentiation. In: *Ontogeny of Hematopoiesis Aplastic Anemia*, Gluckman E and Coulombel L (eds), pp. 209–213. John Libbey Eurotext:

Huss R, Myerson DH and Deeg HJ (1997) Hematopoietic progenitors transfected with a differentiation antigen show cellular transformation and tumour growth in mice. *Int J Exp Pathol* **78**: 177–185

Kamp A (1995) Cell-cycle regulators and cancer. *Trends Genet* **11**: 136–140

Lane PJL, McConnell FM, Schieven GL, Clark EA and Ledbetter JA (1990) The role of MHC class II molecules in human B cell activation. *J Immunol* **144**: 3684–3693

Li Y, Jenkins CW, Nichols MA and Xiong Y (1994) Cell cycle expression and p53 regulation of the cyclin-dependent kinase inhibitor p21. *Oncogene* **9**: 2261–2265

Liu M, Lee MI, Cohen M, Bommakanti M and Fredman LP (1996) Transcriptional activation of the cdk inhibitor p21 by vitamin D3 leads to the induced differentiation of the myelomonocytic cell line U937. *Genes Dev* **10**: 142–147

Luo Y, Hurwitg J and Massague J (1995) Cell cycle inhibition by independent CDK and PCNA binding domains in p21 cip-1. *Nature* **375**: 159–161

Miller AD and Rosman GJ (1989) Improved retroviral vectors for gene transfer and expression. *Bio Techniques* **7**: 980–990

Nourse J, Firpo E, Flanagan WM, Coats S, Polyak K, Lee MH, Massague J, Crabtree GR and Roberts JM (1994) Interleukin-2-mediated elimination of p27 kip-1 cyclin-dependent kinase inhibitor prevented by rapamycin. *Nature* **372**: 570–572

Roberts JM, Koff A, Polyak K, Firpo E, Collins S, Ohtsubo M and Massague J (1994) Cyclins, CDKs and cyclin kinase inhibitors. CSH Symposia on Quantitative Biology, Vol. LIX: 31

Rubin AL, Arnstein P and Rubin H (1990) Physiological induction and reversal of focus formation and tumorigenicity in NIH 3T3 cells. *Proc Nat Acad Sci USA* **87**: 10005–10011

Sher CJ and Roberts JM (1995) Inhibitions of mammalian G1 cyclin-dependent kinases. *Genes Dev* **9**: 1149–1154

Shin SI, Fredman VH, Risser R and Pollack R (1975) Tumorigenicity of virus-transformed cells in nude mice is correlated with anchorage independent growth in vitro. *Proc Natl Acad Sci USA* **72**: 4435–4441

Tavassoli M and Friedenstein A (1983) Hematopoietic stromal microenvironment. *Am J Hematol* **15**: 195–201

Waga S, Hannon GJ, Beach D and Stillman B (1994) The p21 inhibitor of cyclin-dependent kinases controls DNA replication by interaction with PCNA. *Nature* **396**: 574–576

Weissinger EM, Eissner G, Grammer C, Fackler S, Haefner B, Yoon LS, Lu KS, Bazarov A, Sedivy JM, Mischak H and Kolch W (1997) Inhibition of the Raf-1 kinase by cyclic AMP agonists causes apoptosis of v-abl transformed cells. *Mol Cell Biol* **17**: 3229–3241

*© 1999 Cancer Research Campaign*
